# Parental Country of Birth and Socioeconomic Disadvantage in Childhood Overweight and Obesity: Evidence From the Growing Healthy Kids Study

**DOI:** 10.1016/j.focus.2026.100486

**Published:** 2026-02-18

**Authors:** Vilas Kovai, Shanley Chong, Bin Jalaludin, Janice Tang, Shiva Raj Mishra, Michelle Camilleri, Margaret Thomas, Mandy Williams

**Affiliations:** 1 Population Health Service, South West Sydney Local Health District, Liverpool, Australia; 2 School of Medicine, Western Sydney University, Campbelltown, Australia; 3 Prevention Research Collaboration, Charles Perkins Centre, Sydney School of Public Health, The University of Sydney, Camperdown, Australia

**Keywords:** Social class, pediatric obesity, parents, socioeconomic disparities in health

## Abstract

**Introduction:**

SES is a key factor influencing childhood overweight and obesity, but the impact of parents’ country of birth on this link remains uncertain. This study explored how parents’ country of birth affects the association between family SES—measured by area-level disadvantage and annual household income—and childhood obesity.

**Methods:**

Data were obtained from the Growing Healthy Kids population health survey (2018), which included 1,815 children aged 5–16 years. Overweight and obesity were classified using International Obesity Task Force BMI cut offs. SES was assessed through the Index of Relative Socioeconomic Disadvantage and self-reported annual household income. Multinomial mixed-effects models calculated the ORs and 95% CIs for overweight and obesity, stratified by parental country of birth (Australian born versus overseas born), while adjusting for demographic and behavioral covariates.

**Results:**

Children from the most disadvantaged areas faced a higher risk of obesity, regardless of whether their parents were Australian born (OR=1.83 [99% CI=1.70, 1.97]) or overseas born (OR=1.57 [99% CI=1.45, 1.70]). Children of Australian-born parents with household income <$52,000 also experienced a higher risk: OR=3.36 (99% CI=3.06, 3.52). Results for overweight status varied across SES measures and country of birth groups. Children of parents born in Australia and living in moderately disadvantaged areas had a higher risk of being overweight (OR=1.36, 99% CI=1.29, 1.49), whereas those living in the most disadvantaged areas had a lower risk. Children of parents born overseas and living in the most and moderately disadvantaged areas had a higher risk of being overweight (OR=1.19, 99% CI=1.08, 1.32; OR=1.42, 99% CI=1.30, 1.62). Children of overseas-born parents with a household income of $78000–$103,999 had a higher risk of being overweight (OR=1.23, 99% CI=1.13, 1.35).

**Conclusions:**

SES remains a strong predictor of childhood obesity, regardless of the parents' country of birth status. However, patterns for being overweight differ depending on the SES measure used. Area-level SES measures, such as the Index of Relative Socio-Economic Disadvantage; residents' postcodes; and individual SES measures, such as annual household income, can be incorporated into initial assessments in both clinical and population programs. They can be used to develop area-specific tailored interventions, including targeted health promotion initiatives, case management services, and practical support for those in need.

## HIGHLIGHTS

• Children from low-SES households experienced a greater risk of childhood obesity, whether SES was assessed on the basis of household income or the area that they lived in, regardless of whether their parents were born in Australia or abroad.
• Children living in moderately disadvantaged areas had a higher risk of being overweight in both country of birth groups.
• It is critical to reach out to underprivileged groups in both highly and moderately disadvantaged areas, irrespective of parental country of birth.
• Area-level SES indicators are important in initial assessments for clinical and population health programs, alongside annual household income.

## INTRODUCTION

Childhood obesity is a global public health problem, with notable health, economic, and social effects.^1^ The rate of childhood obesity across the globe remains high, with substantial consequences, both immediate and long term.^1^^,^^2^ In 2022, over 20% of children and adolescents aged 5–19 years were classified as overweight, with 8% of children identified as obese.^1^ About a quarter of Australian children are overweight or obese, with no sign of the prevalence decreasing.^3^

The causes of childhood overweight and obesity are complex, involving a mix of genetic, environmental, cultural, behavioral, and socioeconomic factors.^1^^,^^4^ Globally, low SES is a key factor influencing the rate of childhood obesity.^5^ The persistent link between low SES and higher rates of overweight and obesity has been reported in several studies focusing on Australian youth^6^^,^^7^ as well as in children from other high-income countries.^8^ Australian population data show that differences in SES are related to the individual’s country of origin,^9^ which is of particular significance in Australia, where about a third of the population is born overseas or has overseas-born parents.^10^

There has been some progress in investigating obesogenic factors in children and other vulnerable populations; however, studies remain few, and results are inconsistent.^11^ Parents’ country of birth (COB) and migration status^12^^,^^13^; mothers’ perceptions of healthy body weight^14^; maternal BMI^13^; and a range of SES indicators, including parents’ educational level, household income, place of residence, lifestyle patterns, and employment status, are essential contributing factors to childhood overweight and obesity.^15^^,^^16^ Although previous research^9^^,^^17^ has found that both SES and ethnicity are associated with childhood obesity in Australian primary school children, less clear is the extent to which parents’ COB modifies the relationship between SES (measured by both area-level disadvantage and individual household income) and childhood overweight or obesity.

However, it is crucial to examine the role of parents’ COB on childhood obesity. Evidence indicates that factors contributing to childhood obesity are complex, and children in certain regions face a higher risk of becoming obese.^1^^,^^18^ These factors may include cultural beliefs and practices; for example, immigrant parents often hold different health beliefs and dietary habits as well as physical activity (PA) routines across cultures, which can affect their children’s lifestyle choices.^19^ The socioeconomic position of immigrants in the host country is another critical factor associated with childhood obesity.^19^, ^20^, ^21^ This can affect a parent’s ability to create a healthy family environment, impacting children’s physical, psychological, and social growth.^21^ The economic development level of immigrants' countries of origin^22^ and the obesogenic environment in the host country are additional system-level factors that contribute to unhealthy lifestyles, such as the availability of abundant, inexpensive, energy-dense, nutrition-poor foods.^23^

The high rates of childhood overweight and obesity in Australia—especially considering that one third of children have obesity in the South Western Sydney Local Health District (SWSLHD) region of New South Wales (NSW)^24^—have profound implications for public health policy. This includes determining which groups of children should be prioritized for health interventions. The SWSLHD region has a population of over 1 million, including the largest population of culturally and linguistically diverse communities in NSW, with 36% of residents born overseas and approximately 49% of families speaking a language other than English at home.^25^ These demographic features make this region an ideal location for this study.

The objectives of this paper are twofold: (1) to examine the relationship between parents’ SES and childhood overweight and obesity in SWSLHD and (2) to assess whether parents’ COB influences this relationship.

## METHODS

### Study Population

The authors analyzed 1,815 children aged 5–16 years living in South Western Sydney, Australia, using data from the “2018 Growing Healthy Kids-Population Health Survey” (GHK-SWS) to address the study objectives. The GHK-SWS baseline study, a cross-sectional population-based survey of parents or carers of children aged 5–16 years in southwestern Sydney, NSW, Australia, was conducted in 2018, with a follow-up study planned for 2027.

Two papers from the GHK-SWS study provide a detailed overview of the study design and methodology.^26^^,^^27^ In brief, the baseline questionnaire was developed using validated questions from the 2018 NSW Child Population Health Survey,^28^ the NSW Schools Physical Activity and Nutrition Survey,^29^ and the Make Healthy Normal campaign evaluation.^30^ Survey domains included demographics, health status, parental perceptions of height and weight, dietary intake (including fruits, vegetables, and discretionary foods), nutrition-related behaviors, PA participation and sedentary behavior (SB), and several questions on knowledge and attitudes. The questionnaire was translated into Arabic, Vietnamese, and simplified Chinese (Cantonese/Mandarin), because these are the major language groups in this study area. GHK-SWS study data were collected using a computer-assisted telephone interview system.

The target population included all households with children aged 5–16 years within the study area. The sampling frame comprised residents’ landlines and mobile numbers. A sex- and age-matched sample of 1,815 respondents was recruited and included in the GHK-SWS baseline survey. This sample was calculated on the basis of a power of 0.8, an alpha of 5%, and an estimated baseline prevalence of childhood overweight and obesity at 28.4% in residents of South Western Sydney.^25^ The primary caregiver, either mother, father, or grandparents, who was most able to provide information about the selected child was interviewed from each randomly chosen household. After contact, interviewers collected data on the number of eligible children living in the household, along with their sex and ages. If there was more than 1 eligible child, the interviewer randomly selected 1 using the computer-assisted telephone interview system’s random number generator.

### Measures

The study’s primary outcome measure was the child’s BMI. BMI was categorized using the age- and sex-specific International Obesity Task Force definitions^31^: thin/underweight (BMI <18 kg/m^2^), healthy weight (18 kg/m^2^ ≤ BMI < 25 kg/m^2^), overweight (25 ≤ BMI < 30 kg/m^2^), and obese (BMI ≥30 kg/m^2^). To determine BMI, the respondent was asked 2 questions: (1) *How tall is <CHILDNAME> without shoes?* (in centimeters) and (2) *How much does <CHILDNAME> weigh without clothes or shoes?* (in kilograms). Because this paper focused on childhood overweight and obesity, children with a BMI <18 kg/m^2^ were excluded from the analysis (*n*=224).

The study’s main predictor of interest was SES, assessed using area-level SES and participants' household income. At the postcode level, area-level SES was calculated from the 2016 Index of Relative Socio-Economic Disadvantage (IRSED).^32^ The Australian Bureau of Statistics developed the IRSED to compare social and economic disadvantage across geographic areas in Australia. The index is based on census variables, including income, educational attainment, unemployment, and the proportion of the population employed in unskilled jobs. The IRSED was divided into tertiles (Tertile 1: most disadvantaged/low SES, Tertile 2: middle disadvantaged/middle SES, and Tertile 3: least disadvantaged/high SES). The participants' annual household income (before tax) was categorized into 4 groups: $0–$51,999; $52,000–$77,999; $78,000–$103,999; and ≥$104,000.

Sociodemographic variables reported in the survey included both the child’s (age and sex) and parents’ demographic characteristics (educational attainment, annual household income, and rurality of residence), using the Accessibility/Remoteness Index of Australia Plus. The rurality of residence measures remoteness on the basis of travel distance to different-sized population-adjusted service centers.^33^ The Accessibility/Remoteness Index of Australia Plus scores are categorized into 5 groups: major cities, inner regional, outer regional, remote, and very remote.

In addition, because of the known links between healthy lifestyle behaviors, childhood BMI, and area-level SES,^7^^,^^20^ 3 health behavior factors were explored as potential confounders: PA levels, sedentary screen time behaviors (SB), and consumption of sugar-sweetened beverages (SSB). For PA, children were classed on the basis of whether they met the current Australian PA guidelines^34^ (yes, no) and were asked the following question: *Over the past 7 days, on how many days was <CHILDNAME> engaged in moderate to vigorous PA for at least 60 minutes each day (this can be accumulated over the entire day, for example in bouts of 10 minutes)?* For SB, the current Australian guidelines recommend that children aged 5–17 years limit sedentary recreational screen time (such as watching TV, using an iPad, digital versatile/video discs, or a mobile phone) to 2 hours per day.^34^ SB was assessed by asking participants, *In a typical week (Monday to Sunday), how many hours in total does <CHILDNAME> spend watching TV/iPad/digital versatile/video discs, using the computer/iPad/mobile phone for fun or playing video games?* and categorized into <2 hours of screening time per day and >2 hours of screening per day. SSB consumption was assessed by asking participants, *How many cups of soft drink/cordial/sport drink/energy drink does their child usually drink in a day?* Total SSB consumption per day was categorized as no cups, ≤1 cup, or >1 cup. The current Australian Dietary Guidelines discourage the consumption of any SSBs in children owing to the numerous detrimental health effects.^35^

The other variable of interest was the parents’ COB, categorized as Australian born or overseas born. To determine COB, the respondent was asked, *Can you tell me in which country <CHILDNAME>'s mother/father/you was/were born?* Because the authors focused solely on parents (mothers and fathers) as the children's primary caregivers, other care relationships (e.g., grandparents or relatives) were excluded from the analysis (*n*=51). In this paper, parents refer to mothers (73.5%) and fathers (21.5%), and only their responses to survey questions were included in the analysis.

### Statistical Analysis

**Figure 1:**
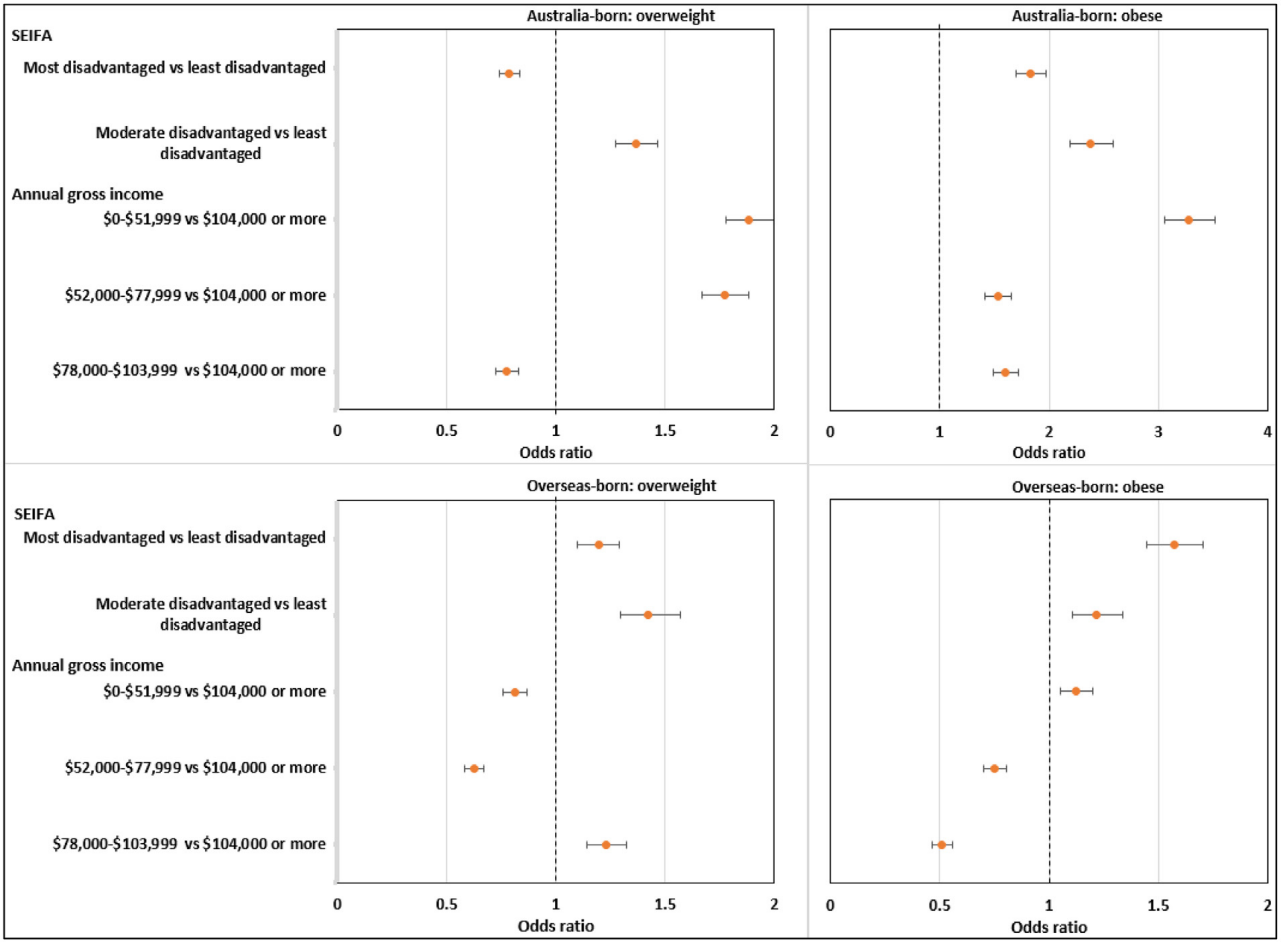
Association between IRSD, annual gross household income, and BMI by parents’ country of birth after adjusting for confounders,^1^ adjusted OR 95% CI. *Note*: Analysis was adjusted for age, sex, parents’ relationship with child (mother or father) and educational attainment, geographic location, sugar-sweetened beverages consumption, whether they met physical activity guideline, and whether they met sedentary behavior guideline, as covariates. IRSD, Index of Relative Socio-Economic Disadvantage.

Descriptive analysis used means (±SD) and proportions to explore participants' sociodemographic and other characteristics, which are presented in Table 1. After the descriptive analysis, univariable associations between the child’s and parent’s characteristics, SES at both the household and area levels, and children’s overweight and obesity were analyzed (adjusted Table 2 and adjusted Figure 1).

**Table 1:** Participants' Sociodemographic Characteristics (N=1,535)

Variable	Level	*n;* %
Age	5–11 years	890; 58.0
	12–16 years	645; 42.0
Sex	Boys	790; 51.2
	Girls	745; 48.8
Parent’s relationship	Mother	1,096; 73.5
	Father	439; 26.5
Parent’s country of birth (missing=45)	Australia	780; 50.7
	Other	755; 49.3
Parent’s educational attainment (missing=7)	≤12 years	430; 33.1
	Trade, TAFE, or diploma	523; 32.0
	University degree	575; 34.9
Annual household Income (before tax) (missing=232)	$0–$51,999	354; 34.2
	$52,000–$77,999	254; 20.5
	$78,000–$103,999	250; 16.3
	$104,000 or more	445; 28.9
Location	Major cities	1,404; 91.7
	Inner regional	131; 8.3
Area-level SES	Most disadvantaged	843; 57.0
	Moderate disadvantaged	263; 16.6
	Least disadvantaged	429; 26.4
BMI	Healthy weight	878; 57.2
	Overweight	367; 22.5
	Obese	290; 20.3
Met physical activity guideline	No	1,212; 77.7
	Yes	323; 22.3
Met sedentary behavior guideline (missing=17)	No	349; 22.2
	Yes	1,169; 77.8
Sugar-sweetened beverages (per day)	No cups	786; 52.4
	1 cup	510; 32.4
	≥2 cups	239; 15.2

TAFE, Technical and Further Education.

**Table 2:** Distributions of Overweight and Obesity by Parent Specific Factors

Variable	Level	Healthy weight	Overweight	Obesity
		*n* (%)	*n* (%)	*n* (%)
Age	5–11 years	483 (53.0)	191 (20.6)	216 (26.4)
	12–16 years	395 (63.0)	176 (25.1)	74 (11.9)
Sex	Boys	435 (55.6)	201 (22.4)	154 (22.0)
	Girls	443 (58.9)	166 (22.6)	136 (18.5)
Parent’s relationship	Mothers	643 (47.8)	254 (22.4)	199 (19.9)
	Fathers	235 (55.6)	113 (22.8)	91 (21.5)
Parent’s country of birth	Australia	466 (60.1)	185 (22.5)	129 (17.4)
	Other	412 (54.2)	182 (22.4)	161 (23.3)
Parent’s educational attainment	≤12 years	219 (51.3)	112 (23.4)	99 (25.3)
	Trade, TAFE, or diploma	305 (57.8)	116 (21.5)	102 (21.0)
	University degree	351 (62.9)	138 (22.4)	86 (14.8)
Annual household Income	$0–$51,999	175 (46.6)	86 (22.8)	93 (30.6)
	$52,000–$77,999	146 (60.0)	58 (22.9)	50 (17.2)
	$78,000–$103,999	145 (61.4)	62 (22.4)	43 (16.2)
	$104,000 or more	271 (62.9)	108 (21.3)	66 (15.8)
Location	Major cities	800 (56.5)	338 (22.9)	266 (20.6)
	Inner regional	78 (65.1)	29 (17.6)	24 (17.3)
Area-level SES	Most disadvantaged	454 (53.6)	209 (22.6)	180 (23.8)
	Middle disadvantaged	147 (56.5)	67 (23.8)	49 (19.6)
	Least disadvantaged	277 (65.4)	91 (21.5)	61 (13.1)
Met physical activity guideline	No	686 (56.4)	301 (24.3)	225 (19.4)
	Yes	192 (60.1)	66 (16.4)	65 (23.5)
Met sedentary behavior guideline	No	181 (51.2)	95 (24.5)	73 (24.4)
	Yes	686 (58.7)	270 (22.2)	213 (19.1)
Sugar sweetened beverages (per day)	No cups	481 (60.3)	171 (21.6)	134 (18.1)
	>0 to <1 cup	287 (58.6)	124 (22.2)	99 (19.2)
	≥1 cups	110 (43.5)	72 (26.2)	57 (30.3)

TAFE, Technical and Further Education.

The variance inflation factor was used to assess multicollinearity. The variance inflation factor values ranged from 1.0 to 2.4, indicating low collinearity among the predictors of interest. The authors conducted generalized linear multinomial mixed models^36^ using the Proc GLIMMIX procedure in SAS, Version 9.4. The models included random intercepts to account for local government area clustering and to explore the associations between area-level SES and children’s overweight and obesity. A compound symmetry covariance structure was selected on the basis of the Akaike Information Criterion. Children’s age, sex, parents’ relationship to the child (as mother or father), parents’ educational attainment, annual household income, and rurality of residence were included as confounders in the final model. Effect modification by the primary carer’s (mother or father) COB was tested and was found to be statistically significant (*p*<0.0001). Results of the adjusted multinomial multilevel regression model are presented as ORs with 95% CIs. A Bonferroni adjustment was applied for stratified analyses by the primary carer’s COB, along with a 99% CI. Survey weighting was incorporated in all analyses to ensure that the survey sample was representative of all children living in SWSLHD. Missing data were excluded from the analysis. The analysis was conducted using SAS (Version 9.4).^36^

This study was approved by the Human Research Ethics Committee of SWSLHD (HE18/078), which covered both primary and secondary analysis. Before the interviews, informed verbal consent was obtained from all the study participants (i.e., parents or carers aged ≥18 years who could provide the maximum information about the selected child). The data were collected in October and November 2018. It was accessed for research purposes in February 2021, and the authors had no access to information that could identify participants during or after data collection. All information obtained in connection with this study was kept confidential, and the data were deidentified before analysis and reporting. All study materials were stored in line with the Human Research Ethics Committee’s guidelines.

## RESULTS

The sociodemographic characteristics of participants are outlined in Table 1. The GHK-SWS study collected data from 1,815 children. After excluding thin (underweight) children (*n*=224) and other nonparents (*n*=51), the analysis is based on 1,535 children. Overall, 57% of the children were within the healthy weight range (*n*=878), 23% were overweight (*n*=367), and 20% were obese (*n*=290). About half of the participants were boys (51%, *n*=790), with a mean age of 10.8 (SD=4.5) years. More than half (57%) lived in disadvantaged areas, and approximately one third (34%) reported an annual household income of less than $52,000. Most parents were mothers (74%), of whom about half (51%) were born in Australia. Approximately 35% had completed a university degree, 32% completed a Technical and Further Education diploma, and 33% had received ≤12 years of school education. The distribution of overweight and obesity by parent-specific characteristics is presented in Table 2.

In an adjusted model, using healthy weight as the reference, the sociodemographic factors associated with higher odds of being overweight in children included girls; aged 5–11 years; parents born in Australia; parents with only 12 years or less of education; families reporting an annual household income below $77,999; and residing in a major city and a moderately disadvantaged area (Table 3). Children were more likely to be obese if they were boys; were aged 5–11 years; had fathers as the primary carers; had parents born overseas; had parents who did not complete a university degree; came from families with an annual household income of less than $52,000; lived in inner regional areas; or lived in the most disadvantaged areas (Table 3).

**Table 3:** Adjusted Multinominal Regression Between Sociodemographic Factors, Health Behaviors, and Overweight and Obesity^a^

Variable	Level	AOR (95% CI)	AOR (95% CI)
Age	5–11 years	ref	ref
	12–16 years	0.83 (0.81, 0.86)	0.28 (0.27, 0.29)
Sex	Boys	0.98 (0.95, 1)	1.04 (1.01, 1.07)
	Girls	ref	ref
Parent’s relationship	Mothers	ref	ref
	Fathers	0.99 (0.96, 1.02)	1.18 (1.14, 1.22)
Parent’s country of birth	Australia	ref	ref
	Other	0.88 (0.85, 0.91)	1.43 (1.38, 1.48)
Parent’s educational attainment	≤12 years	1.1 (1.06, 1.14)	1.99 (1.91, 2.08)
	Trade, TAFE, or diploma	1.02 (0.99, 1.06)	1.49 (1.43, 1.55)
	University degree	ref	ref
Annual household Income	$0–$51,999	1.3 (1.24, 1.35)	1.66 (1.59, 1.74)
	$52,000–$77,999	1.09 (1.05, 1.14)	0.92 (0.88, 0.97)
	$78,000–$103,999	1 (0.95, 1.05)	0.93 (0.88, 0.98)
	$104,000 or more	ref	ref
Location	Major cities	ref	ref
	Inner regional	0.68 (0.62, 0.75)	3.11 (2.83, 3.43)
Area-level SES	Most disadvantaged	0.95 (0.91, 0.99)	1.49 (1.42, 1.57)
	Middle disadvantaged	1.27 (1.21, 1.34)	1.74 (1.63, 1.84)
	Least disadvantaged	ref	ref
Met physical activity guideline	No	1.42 (1.36, 1.47)	0.92 (0.89, 0.96)
	Yes	ref	ref
Met sedentary behavior guideline	No	1.13 (1.09, 1.17)	1.49 (1.44, 1.55)
	Yes	ref	ref
Sugar sweetened beverages (per day)	No cups	ref	ref
	>0 to <1 cup	1.22 (1.18, 1.26)	1.37 (1.32, 1.42)
	≥1 cups	1.52 (1.45, 1.59)	2.52 (2.42, 2.63)

a Type 3, *p*-value for each variable is <0.0001. TAFE, Technical and Further Education.

In the same adjusted model (Table 3), children are more likely to be overweight when the PA and SSB guidelines are not met. Similar patterns were observed for children with higher odds of being obese, except for meeting PA guidelines: children who meet PA guidelines are more likely to be obese (OR=1.09, 95 % CI=1.04, 1.12) than all other children.

Figure 1 shows the associations between area-level SES, annual gross household income, and child overweight and obesity by parents’ COB after adjusting for confounders.

The odds of a child being overweight are highest if their parents were born in Australia and reside in moderately disadvantaged areas (OR=1.36, 99% CI=1.29, 1.49) and lowest if their parents live in the most disadvantaged areas (OR=0.78, 99% CI=0.71, 0.86) compared with families in the least disadvantaged areas.

The odds of a child being overweight are highest if their parents were born in Australia and have a yearly household income below $77,999 ($0–$51,999: OR=1.88, 99% CI=1.81, 2.04; $52,000–$77,999: OR=1.77, 99% CI=1.69, 1.92), whereas an annual household income of $78,000–$103,999 was associated with a lower risk (OR=0.78, 99% CI=0.69, 0.84) than for families with household income above $104,000.

The odds of a child being obese were higher among participants living in the most and moderately disadvantaged areas (most disadvantaged areas: OR=1.83, 99% CI=1.66, 2.02; moderately disadvantaged areas: OR=2.38, 99% CI=2.13, 2.65) than among those living in the least disadvantaged areas.

The odds of being obese were also higher across all 3 annual household income categories than the reference category of >$104,000: ($0–$51,999: OR=3.28, 99% CI=2.99–3.60; $52,000–$77,999: OR=1.53, 99% CI=1.38–1.70; $78,000–$103,999: OR=1.60, 99% CI=1.45, 1.76).

The odds of a child being overweight are highest among children whose parents were born overseas and who live in the most disadvantaged areas (OR=1.19, 99% CI=1.08, 1.32) and in moderately disadvantaged areas (OR=1.42, 99% CI=1.30, 1.62) than among children living in the least disadvantaged areas.

The odds of children being overweight were lower if their parents were born overseas and had a yearly household income below $77,999 ($0–$51,999: OR=0.81, 99% CI=0.72, 0.88; $52,000–$77,999: OR=0.62, 99% CI=0.53–0.69). However, the odds of children being overweight were higher among families with household annual income of $78,000–$103,999 (OR=1.23, 99% CI=1.13, 1.35) than among families with household income above $104,000.

As area-level SES increased, the odds of these children being obese increased sharply from the least disadvantaged to the most disadvantaged areas (moderately disadvantaged areas: OR=1.22, 99% CI=1.09, 1.38; most disadvantaged areas: OR=1.57, 99% CI=1.45, 1.75).

The odds of children being obese were slightly higher among families with a reported annual household income of $0–$51,999 (OR=1.12, 99% CI=1.04, 1.22), although to a much smaller extent than in children whose parents were born in Australia. Conversely, the odds of children being obese were lower among participants whose annual household income was between $52,000 and $103,999 ($52,000–$77,999: OR=0.75, 99% CI=0.66, 0.82; $78,000–$103,999: OR=0.51, 99% CI=0.40, 0.57).

Further analyses were conducted among carers born overseas, categorized by continent of birth according to the Standard Australian Classification of Countries: Asia (*n*=287), the Middle East (*n*=183), sub-Saharan Africa and Europe (*n*=120), and others (*n*=165). Others included the Americas (*n*=56), Oceania (*n*=92), and other continents (*n*=17). The impact of SES across carer’s continents of birth was generally consistent, except for those born in other continents, where a strong positive impact of household annual income on the risk of children being overweight was observed ($0–$51,999: OR=8.28, 99% CI=6.56, 10.47; $52,000–$77,999: OR=10.22, 99% CI=8.08, 12.94; $78,000–$103,999: OR=26.69, 99% CI=19.75, 30.87).

## DISCUSSION

This study found a significant association between SES measures (assessed by area-level disadvantage and annual household income) and the risk of overweight and obesity among children aged 5–16 years living in Southwest Sydney, the most culturally diverse region in Australia. The obesity findings are consistent across SES metrics and COB groups. Children from low SES households faced a higher risk for childhood obesity, whether SES was measured by household income or area of residence, and this association remained regardless of whether the responding parent was born in Australia or overseas. The risk of childhood obesity was 3 times higher among the Australian-born parents with the lowest household income.

However, overweight results vary across SES and COB groups if parents are living in the most disadvantaged areas. For instance, children of Australian-born parents living in highly disadvantaged areas had the lowest risk of being overweight. Conversely, children of overseas-born parents in similar areas faced a higher risk. The overweight results are consistent across SES and COB groups when parents live in moderately disadvantaged regions. For instance, the children of parents living in moderately disadvantaged areas are at a higher risk of being overweight, regardless of their parents' COB status.

When SES was measured using annual household income, the opposite pattern was observed. Children of Australian-born parents with yearly low household incomes had a higher risk of being overweight. In contrast, children of overseas-born parents with low annual household incomes had a lower risk of being overweight.

The increased risk of obesity among children from low SES backgrounds across both Australian- and overseas-born parents in this study aligns with the recent WHO report on ending childhood obesity.^37^ The existing evidence from the WHO and recent Australian study shows that the risks of childhood obesity are highest in lower SES groups living in high-income countries.^38^^,^^39^ Indeed, in high-income countries, children from low SES populations, such as immigrants, indigenous people, and culturally and linguistically diverse groups, are at a particularly high risk of developing obesity.^37^

Although many studies have examined the contribution of SES to differences in obesity across ethnic groups, very few have considered migration status as a factor.^40^ The limited evidence from studies conducted in Europe,^41^ the U.S.,^42^ Australia,^43^ and Germany^44^ suggests that weight increases substantially among migrant children over a postmigration period of 10–15 years after migration, and the social and economic disadvantage faced by migrants is linked to an increased risk of childhood obesity, an association this study findings confirm.

In this study, the influence of SES, measured by both area and household income, on childhood overweight and obesity was inconsistent across the 2 measures. SES, measured by household income and area-level disadvantage, provides different contextual information. In addition, the risk of childhood overweight and SES is also affected by the specific SES measure used in the analysis. For instance, children of immigrants with low SES faced a higher risk of being overweight when parental education was used as a proxy for SES,^44^ but not when employment was used as an indicator of SES.^45^ Area-level SES also captures other area-level characteristics such as access to healthy foods and facilities for PA, employment opportunities, and crime rates. Consequently, although household income is a vital SES metric, area-level SES may be a more effective measure for resource allocation or for implementing population-level programs. This study, along with the existing literature, highlights the need for further detailed research to better understand the complexity and variation of childhood overweight and obesity among different population subgroups.

Although household income is a known factor associated with childhood obesity, this link was significantly influenced by parents’ COB. A systematic review of socioeconomic inequalities in childhood obesity in the United Kingdom, for example, found that although household income is a reliable determinant of childhood obesity, results varied across studies.^46^

This research also confirms that low household income is consistently associated with childhood obesity, regardless of children's COB status. However, when it comes to the question of being overweight, parents’ COB acts as a modifying factor. Future studies would benefit from including both area- and individual-level SES measures to better understand their association with childhood overweight and obesity.

Childhood overweight and obesity arise from exposure of the child to an obesogenic environment and insufficient responses to that environment. Such responses vary from person to person for reasons that may be social, cultural, economic, environmental, or behavioral, as discussed below.

There are plausible explanations for the increased risk of childhood overweight and/or obesity among children of overseas-born parents living in disadvantaged neighborhoods. For instance, residents of such areas are more likely to have low wages or face unemployment or insecure employment, especially if they are recent migrants, are women, are single parents with dependent children, or speak a language other than English at home.^47^^,^^48^ Subsequent generations of migrants, even those with higher income levels, are also more prone to marginalization and fewer opportunities,^49^ often achieving lower educational levels, and lack resources to own property.^50^ In addition, although migrants may arrive in their new country with certain health advantages, they are more likely to experience social isolation, marginalization, stress, and weight gain 10–15 years after migration.^40^

Apart from the socioeconomic disadvantage faced by migrants, their exposure to obesogenic environmental factors in host countries may also contribute to the higher prevalence of overweight and obesity among their children.^19^^,^^51^ This phenomenon has been linked to the challenges of acculturation, including lifestyle changes in the host country, lower levels of PA, and increased SB^25^^,^^41^ and the adoption of unhealthy diets.^51^, ^52^, ^53^

The positive association between household income and carers' COB highlights the complex interaction between family environment and parental food habits, particularly among families in the Oceania and American regions with higher disposable income (e.g., earning $78,000–$103,999 per year). For instance, the American region has the highest rates of overweight and obesity worldwide, with 67.5% of adults being overweight or obese.^54^ Some areas within the Oceania region have obesity rates 5 times that of the world average.^55^ Carers’ BMI and cultural backgrounds could offer additional insights into how their COB influences the risk of children becoming overweight or obese.

The results revealed an unexpected finding that some children, despite meeting PA guidelines, were obese. This suggests that the complex interaction between family environment and parental food habits shapes children’s BMI status. Evidence suggests that the family environment plays a crucial role in shaping a child's healthy lifestyle, including limiting screen time, emphasizing active transportation, ensuring adequate PA, promoting a nutritious diet, and ensuring sufficient sleep.^56^ The beliefs and practices of parents and carers are underlying factors that influence parenting style and help create a healthy family environment.^56^

### Limitations

This study has some limitations. BMI calculated from self-reported height and weight is prone to measurement bias. However, evidence from a previous study suggests that correlations between self-reported and objectively measured height, weight, and BMI remained high, thereby supporting the use of self-reported values.^57^ Evidence indicates that self-reported weight and height in children can be a reliable measure when the data are obtained from a population-representative, large sample.^58^, ^59^, ^60^ Although the study provides comprehensive information on the basis of a sufficient sample size, representative population data, and a standardized questionnaire, a potential limitation is the inability to assess the causal association between SES, parents’ COB, and childhood overweight and obesity owing to its cross-sectional design. Although children’s health behaviors, such as meeting PA, SB, and SSB consumption guidelines, were adjusted in the final model, future research that includes data on carer’s BMI and cultural differences between carer’s countries of birth and other obesogenic factors (such as the availability of unhealthy food stores) would be valuable area for further investigation.

## CONCLUSIONS

Children from low SES households faced a higher risk for childhood obesity, whether SES was measured by household income or area of residence, and this association remained regardless of whether the responding parent was born in Australia or overseas. Children residing in moderately disadvantaged areas faced a higher risk of being overweight across both COB groups. Public health efforts should target underprivileged groups in both highly and moderately disadvantaged regions, regardless of parental COB. Recognizing SES-related factors across all COB groups can inform the development of culturally appropriate obesity prevention strategies. Area-level SES indicators, such as the IRSED or residents’ postcodes, alongside annual household income, should be included in initial assessments for both clinical and population health programs. This data may be vital for understanding barriers to care and for creating tailored interventions, including targeted health-promotion initiatives and practical support for those in need. Future research should integrate both area-level and household-level SES measures to better understand the socioeconomic factors influencing childhood obesity.
